# The role of health in achieving the sustainable development goals

**DOI:** 10.2471/BLT.18.221432

**Published:** 2018-09-01

**Authors:** Shambhu Acharya, Vivian Lin, Natasha Dhingra

**Affiliations:** a Department of Country Cooperation and Collaboration with the UN System, World Health Organization, avenue Appia 20, 1211 Geneva 27, Switzerland.; b Division of Health Systems, World Health Organization Regional Office for Western Pacific, Manila, Philippines.

*Transforming our world: the 2030 agenda for sustainable development* outlines a transformative vision with 17 sustainable development goals (SDGs) for economic, social and environmental development.^1^ While only SDG 3, to ensure healthy lives and promote well-being for all at all ages, focuses on human health, all goals are interrelated. This issue of the *Bulletin of the World Health Organization* examines the relationship between health and the SDGs.

Implementing the 2030 agenda requires a multistakeholder, multi-actor response. Innovations and development in policy, technology and research must include dialogue between governments, the private sector, civil society organizations and nongovernmental organizations; most importantly, strong community involvement is needed.^2^ The focus of the 2030 agenda on addressing country-level needs is based on the engagement of all actors and sectors, as opposed to the traditional top-down, single-sector approach. Intersectoral governance is at the core of how to approach the social determinants of health,^3^ and how to address antimicrobial resistance through a one health approach.^4^

More specifically, to reach target 3.8, to achieve universal health coverage, Member States must ensure access to health services by introducing protection against catastrophic health expenditure.^5^ Obstacles and financial hardships associated with weak health systems and inadequate financing mechanisms not only exacerbate health inequities, but also jeopardize the achievement of other SDGs.^6^ For instance, catastrophic health spending^7^ has pushed almost 100 million people into poverty annually, while leaving over half of the world’s population without access to essential health services.^5^ Most out-of-pocket payments, the cause of catastrophic expenditure, arise from high medication costs, which are likely to increase in line with the global increase in noncommunicable diseases and the need for long-term treatment. 

Universal health coverage could therefore contribute to achieving the SDGs by producing equitable and sustainable health outcomes. Many health disparities between people with different socioeconomic status are compounded by gaps in good governance. For instance, corruption makes access to health services, medication and information much more difficult for the vulnerable.^6^ In addition, factors such as ethnicity, gender and disability can further exacerbate these health disparities.^8^ Hence, tracking indicators that measure the health of vulnerable groups are essential. Monitoring the status of equitable access to health care could also shed light on the status of human rights and social equality within states.^9^ Those people not receiving adequate health services are probably also disadvantaged in other social aspects.^10^ A better understanding of factors contributing to access to health services will help shape policies to attain SDG 3, and support the achievement of other SDGs such as attaining gender equality, reducing poverty and improving education.^9^^,^^10^

Currently, robust data on health-related targets and indicators is lacking in many countries; developing better implementation and measurement tools, and linking data across sectors, is a cornerstone of the SDGs and should be a priority in global health policy dialogue.^6^^,^^10^^,^^11^

The 2030 agenda also places emphasis on the environmental determinants of health. The 2016 World Health Organization report *Preventing disease through healthy environments* estimated that 23% of all deaths could be attributed to environmental issues such as air pollution, poor sanitation, exposure to radiation and other environment-related causes.^12^ Progress on the SDGs that target environmental improvements will also improve health indicators.

While countries present national voluntary annual reports at the high-level political forum held in New York, national and international development communities still lack efficient and effective data collection mechanisms, to better report on the status of the progress of Member States towards the SDGs. The WHO *Thirteenth General Programme of Work 2019–2023*,^13^ has introduced an impact framework to measure country results and progress in achieving the health-related targets of the SDGs. This framework can assist in ensuring that Member States are able to collect, monitor and report on such progress. The United Nations and its specialized agencies, should work collaboratively to address social, commercial, economic and environmental determinants of health and to strengthen health systems, contributing to the attainment of all SDGs.
